# A glimpse into social perception in light of vitality forms

**DOI:** 10.3389/fpsyg.2022.823971

**Published:** 2022-09-13

**Authors:** Qingming Liu, Jinxin Zhang, Da Dong, Wei Chen

**Affiliations:** ^1^Center for Brain, Mind and Education, Shaoxing University, Shaoxing, China; ^2^Department of Psychology, Shaoxing University, Shaoxing, China; ^3^Department of Psychology, Zhejiang Normal University, Jinhua, China; ^4^Interdisciplinary Center for Philosophy and Cognitive Sciences, Renmin University of China, Beijing, China

**Keywords:** vitality forms, social perception, intention understanding, social affordances, social emotion

## Abstract

The American psychoanalyst and developmental psychologist Daniel Stern’s idea of vitality forms might suggest a new solution to explain how other minds are intensely expressed in their actions. Vitality forms characterize the expressive style of actions. The effective perception of vitality forms allows people to recognize the affective states and intentions of others in their actions, and could even open the possibility of properties of objects that are indicated by the given actions. Currently, neurophysiological studies present that there might be a neural mirror mechanism in the dorso-central insula (DCI), middle cingulate cortex (MCC), and other related cerebral areas, which serve to preferably perceive and deliver vitality forms of actions. In this article, possible types of vitality forms related to other minds, which have been brought to particular attention in recent years, have been collected and discussed in the following four areas: (1) Vitality forms on understanding non-verbal intention, (2) on understanding verbal intention, (3) vitality forms as grounding social cognition, and (4) as grounding social emotion. These four areas, however, might refer to an entirety of a binary actor-observer communicative landscape. In this review, we try to simplify the analysis by relying on two fundamental dimensions of criteria: first, the idea of vitality forms is conceived as the most basic way of observing subsequent higher-order dimensions of action, that is, understanding intention in the style of action. Thus, in the first two subsections, the relationships between vitality forms and their roles in understanding non-verbal and verbal intention have been discussed. Second, vitality forms could also be conceived as background conditions of all the other mental categories, that is, vitality forms can ground cognition and emotion in a social context. In the second dimension, the existence of social cognition or emotion depends on the existence of the stylistic kinematics of action. A grounding relation is used to distinguish a ground, that is, vitality forms, and its grounded mental categories. As relating with the domain of social perception, in this review, it has been discussed vitality forms possibly could ground social cognition and social emotion, respectively.

## Introduction

### Epigraph

The expressiveness of vitality affects can be likened to that of a puppet show. The puppets have little or no capacity to express categories of affects by way of facial signals, and their repertoire of conventionalized gestural or postural affect signals is usually impoverished. It is from the way they move in general that we infer the different vitality affects from the activation contours they trace. Most often, the characters of different puppets are largely defined in terms of particular vitality affects: one may be lethargic, with drooping limbs and hanging head, another forceful, and still another jaunty.

(Stern, 1998, p. 56)

### Introduction

Adults’ affective experiences have been categorized to a large extent. These are, in a certain sense, a result of social life over a period of time. In the categories of affects, happiness and sadness (the two extremes of affective states) would not be confused for a disciplined adult. It seems that each category owns an unambiguous definition and corresponds to a distinct experiential state. When an adult cries, she cries for a purpose, so to speak; more importantly, the purpose can be articulated in a verbal way. When an adult acts, she acts intentionally toward an object, no matter actual or virtual. Then, consider the affective lives of young infants. In contrast, categories of affects in infants are difficult to be recognized clearly and distinctly by an arbitrary external observer. Babies cry loudly and non-verbally; babies act intensely, and in most instances, use their entire body. It seems that there is an explanatory gap between adults’ categories of affects and infants’ somehow undifferentiated primordial feelings along the lines of orthodox affective category theory; representatives of the latter include Charles Darwin (1872).

The American psychoanalyst and developmental psychologist Daniel Stern in a radically different sense disagreed with affective category theory. Stern once summarized the latter’s view,

“…discrete categories of affect…each of these had an innate discrete facial display and a distinct quality of feeling and that these innate patterns evolved as social signals “understood” by all members to enhance species survival” (Stern, 1998, p. 54–55).

On the contrary, Stern founded affective *gestalt* (form) theory, or what he precisely named “vitality affect” and then the final generalized theory, namely “vitality form.” In this transition of concepts (affect → form), Stern wanted to go beyond the mere affective spheres of one category of the human mind. The concept of vitality forms was then conceived by Stern as background conditions of all mental categories (including attention, emotion, volition, etc.). Practically, this idea is based on Stern’s long-term close observation of young infants’ interpersonal world, especially parental–infant dyadic interactions, for example, the affective bonding relations of infants with their mothers (Stern, 1977). A common definition of vitality forms goes like this: this crucial term is directly related to basic kinematic characteristics of actions, which represent lively experiential states of the mind/the agent. Specifically, it refers to how the action is carried out in space and time, that is, which refers to the “how” dimension of the action. Each vitality form has a specific kinematic “contour” which can be detected according to the kinematics of actions. Depending on people’s affective or cognitive states, their actions may take on different styles or manners of vitality forms. For example, the action of gripping a cup can be “strong” or “delicate”; the action of touching a cat can be “rude” or “gentle” (Stern, 2010).

Presently, there is an increasingly influential current in social perception debates that seeks inspiration from Stern’s vitality forms (cf. Di Cesare et al., 2014; Marraffa and Meini, 2019; Casartelli et al., 2020a). The idea of vitality forms also attracts mirror neurons’ discoverer, the neuroscientist Giacomo Rizzolatti’s attention. Through the efforts of Rizzolatti, Di Cesare, and their research team, the issue of neural bases of vitality forms, relating to the neural mirroring mechanism of social perception, is also discussed in the realm at present (cf. Gallese and Rochat, 2018; Di Cesare et al., 2020; Rizzolatti et al., 2021).

Generally, there are three traditional theoretical approaches to the current debates on the philosophy of social perception: Rationality theory, theory-theory, and simulation theory (Apperly, 2010). Rationality theory supposes that each human person is a rational being; reading other minds is a process of actively rationalizing others’ beliefs (Goldman, 2006). Theory-theory argues that people explain and forecast the behavior of others by applying a set of folk psychological theories (Davies and Stone, 1995); thus, the key point of this approach relates to the internal construction of a *theory* of one’s own. Simulation theory assumes that people’s own behaviors and psychological relationships are similar to those of others. Thus, in order to explain the behavior of others, in a certain way, people *simulate* the mental states of others in their own minds (cf. Hurley, 2008). Currently, orthodox views of other minds as indirectly inferred activities have been challenged by direct social perception theory already; that is, it is possible to directly *see* the mental states of other people (cf. Gallagher, 2020; Krueger, 2021). Besides, in recent times, experimental psychologists, such as Cristina Becchio, have already tried to design a series of down-to-earth experiments to defend this new approach (Becchio et al., 2018).

This article proposes that the idea of vitality forms allows people to *see* others’ minds in their actions directly. In other words, it is possible to understand the attitudes or affective states of others by simply observing their actions (e.g., by listening to their varying tone of voice).^1^ Vitality forms represent the way an action could be performed. Imagine an agent interacts with the other agent in a dyad. According to their attitude (positive or negative), interoceptive state (feel good or feel bad), and emotion (happy or sad), the final action is that they will pass an object gently or rudely. Note that during this *very* action, so to speak, there is always a one-to-one correspondence between the intention of the agent (for passing an object) and the how-dimension of action (vitality forms, i.e., how an agent passes this object). Gallagher (2020) claims that the concept of vitality forms possibly is the prerequisite of subsequent contents and intentions of social perception; he highlights that only in this context people can talk about “an emergence of meaning, a meaning that emerges in the interaction itself” (Gallagher, 2020, p. 161; see also Gallagher et al., 2022). Krueger (2021) tries to defend direct social perception with the aid of vitality forms. He argues that people see others’ actions “performed *in a particular sort of way*, as embodying a particular manner or style” (Krueger, 2021, p. S373). That is, people see vitality forms directly, observing the mind directly in action (Krueger, 2021, p. S368). Combining those converging reflections of Gallagher, Krueger, and others, the approach of action-how (vitality forms) is radically different from action-what (contents) and action-why (intentions). In a certain sense, it defends the direct social perception approach (as well as challenges the orthodox mindreading theory). In the following paragraphs, we have collected and reviewed the existing relevant empirical research with a synopsis to clarify how the mental states of others are directly represented in the perception of others’ expressive kinematics through vitality forms of action. The scope of this paper is to reveal the significance of vitality forms for kinematic interpersonal relations.

### Burgeoning areas of vitality forms to social perception

In Stern’s approach, social perception activities take parental–infant dyadic relationships as proto-types. Vitality forms occupy a new dimension apart from affective category theory, actions *per se* convey enough in revealing the kinematic signatures of social perception. To adults, previous parental–infant intersubjective activities may remain components in the adult stage. Vitality forms should be seen as more fundamental than derivative categories of affects, and contents or intentions of mindreading. Thus, more or less metaphorically, not only infants but also adults are immersed in “feelings of vitality”:

“Different feelings of vitality can be expressed in a multitude of parental acts that do not qualify as “regular” affective acts: how the mother picks up baby, folds the diapers, grooms her hair or the baby’s hair, reaches for a bottle, unbuttons her blouse. The infant is immersed in these “feelings of vitality”” (Stern, 1998, p. 54).

Another important person in the realm of developmental intersubjectivity studies, Trevarthen (2013) applauds highly of Stern’s last monograph on forms of vitality, “has made a bible for all humanistic and art therapies” (Trevarthen, 2013, p. 44). In a sense, vitality forms play a constitutive role in the bottom level of the stratified structures of intersubjectivity. Combining Trevarthen’s concept of primary intersubjectivity (the stage of young infants before 9 months), vitality forms are the first perceivable signatures that young infants can *see* (directly) (cf. Gallagher, 2020). However, it is far from clear “how body, brain, and mind collaborate in its shared vitality” (Trevarthen, 2019, p. 31) based on the current empirical studies of vitality forms.

Further, in this article, possible types of vitality forms related to other minds are collected and discussed in the following four areas across two dimensions of criteria, which have been brought to particular attention in recent years: (1) Vitality forms on understanding non-verbal intention, (2) on understanding verbal intention, (3) vitality forms as grounding social cognition, and (4) as grounding social emotion. These four areas might refer to an entirety of a binary actor–observer communicative landscape. Consider that a person performing a certain action is being observed, or, in other words, there exists an ongoing non-verbal/verbal communication between two actors (also as observers). There are five factors that can possibly be considered within this landscape: factors involving the *actor*, factors involving the *observer*, factors involving the *action*, factors involving the *relationship* between the actor and the observer, and factors involving the *context* (Kemmerer, 2021). All intersubjective characteristics of ongoing actions within a dyad can be seen as instantiations and their combinations of these five factors.

Thus, we try to simplify the analysis by relying on two fundamental dimensions: first, the idea of vitality forms is conceived as the most basic way of *seeing* subsequent higher-order dimensions of action, that is, understanding intention in the *style* of action (Stern, 2010; Krueger, 2021). Thus, the following first two subsections discuss the relationships between vitality forms and their role in understanding non-verbal and verbal intentions.

Second, vitality forms could also be conceived as background conditions of all the other mental categories, that is, vitality forms can *ground* cognition and emotion in social context.^2^ In the second dimension, the existence of social cognition or emotion depends on the existence of the stylistic kinematics of action. A grounding relation is used to distinguish a ground, namely vitality forms, and its grounded mental categories (cf. Barsalou, 2010). In relating to the domain of social perception, we discuss vitality forms that possibly could ground social cognition and social emotion, respectively^3^ (see Table 1).

**TABLE 1:** Four burgeoning areas that vitality forms serve as understanding and grounding, respectively.

Vitality forms		
Understanding	Non-verbal intention	Verbal intention
Grounding	Social cognition	Social emotion

The relationships between vitality forms and these four burgeoning areas are briefly reviewed in this article. Increasing evidence suggests that one type of non-inferentialist social perception theory allied with vitality forms promises to support a direct social perception approach. However, this enterprise underlying inferentialist–non-inferentialist social perception debate is still forthcoming because different studies lack essential theoretical relevance until the present time. In a long-range sense, the aim of the article is to make headway on the possible cooperation of vitality forms and direct social perception, that is, seeing the mind in the vitality forms of action directly.

### Vitality forms on understanding non-verbal intention

When an agent has a positive or negative attitude (in other words, affective valence) toward the other agent, they will perform the following actions in a gentle or rude way, regardless of the type of action contents or intentions (i.e., taking a bottle to drink for themselves, or to throw it in the face of others). Each action is characterized by three different aspects: the content (action-what: Taking the bottle), the intention (action-why: Taking the bottle because of thirst), and the vitality forms (action-how: Taking the bottle hastily rather than tardily) (Stern, 2010).^4^ For example, observing a person who greets you in a distance, in a certain sense *via* their action kinematics can help understand if they feel good or not, or if they are happy or sad. The same thing happens when answering the phone. It is possible to comprehend how the other person feels, directly, by hearing the tone contours of their voice regardless of verbal information. In one study specifically related to auditory vitality forms, Di Cesare et al. (2022) imply that communicative intention is a necessary condition for processing one of the candidates of neural bases of vitality forms, that is, dorso-central insula (DCI).

Prior to introducing the term “vitality forms,” several empirical studies in cognitive psychology may somehow confirm the significance of kinematics of behavior in effective communicative interactions. Becchio et al. (2008) show that the kinematics of the movements is sensitive to social intention, and the kinematic patterns of the movements performed under two conditions (social condition and single-agent condition) are significantly different. Sartori et al. (2011) imply that action observers can distinguish between cooperative, competitive, and personal actions simply by observing the initial stretch of the action to the grasp stage; besides, an intention identification study comparing video and point light source clips by Manera et al. (2010) seem to also confirm this conclusion (also see Cavallo et al., 2016; Koul et al., 2016). Cavina-Pratesi et al. (2011) find that skilled magicians are sensitive to kinematic differences between grasping movements of real and imaginary objects. Ansuini et al. (2016) suggest that in a certain sense, the kinematic characteristics of pantomime movements may reflect the weight information of the target object. Podda et al.’s (2017) result shows that the action observer could tell whether a non-existent object is light or heavy on the basis of the kinematics of the observed action. However, it should be noted that all the examples provided in this passage do not assume a direct link between those previous kinematics research studies and vitality forms.

The relationship between autism spectrum disorder (ASD) and perceiving vitality forms has sought attention from an increasing number of researchers (Bystrom et al., 2019). ASD is a neurodevelopmental disorder with various clinical features, including deficits in social skills, verbal and non-verbal communication, and restricted and repetitive behaviors (cf. American Psychiatric Association, 2013, p. 40). There exists evidence that individuals with ASD are poor at recognizing emotional expressions in others (Atkinson, 2009). This may be linked to their difficulty in recognizing the vitality forms of other people’s movements. Rochat et al. (2013) then investigate this hypothesis by asking children with ASD and healthy children to judge whether the two observed movements are similar or different in terms of vitality forms. The results show a distinct separation between the two tasks; autistic children have a remarkable impairment in recognizing the vitality forms of other people’s actions. Casartelli et al. (2020a) compare autistic children with healthy children in different vitality forms (mild or rude) when performing hand movements (for example, placing a bottle, throwing a ball, or giving cookies). The results present those children with ASD show significant difficulty in changing their vitality forms while maintaining the same type of action. That is, they cannot express the vitality forms as a component of action, which is different from the goal of action. Effective social interaction is not only about understanding others, but also about making others understand us. It is a bilateral interaction process. Casartelli et al. (2020b) then further investigate whether healthy children could recognize vitality forms in autistic children. Based on the previous study, they find that healthy children have significant difficulties in understanding vitality forms displayed by autistic children, and yet still perform poorly with the aid of information feedback (for the latest review of ASD studies in the light of vitality forms, see Rochat and Gallese, 2022).

### Vitality forms on understanding verbal intention

In our view, the role of vitality forms in understanding non-verbal intention is of primary significance here. Young infants have poor verbal communicative capacities. The vast majority of the communicative activities within a parental–infant dyad are presented in a non-verbal way. Besides, in activities of verbal communication, adult people to a large degree can ignore those kinematic clues considered here that have no direct relation with semantic information of human speech (such as the pitch, accent, and tone of voice). So, we turn to focus on young infants’ vitality forms of speech, since they depend largely on the how-dimension of action for daily communication and developing their social cognition capacities. Generally, in relation to vitality forms in the realm of social perception, the studies of non-verbal intention and those of verbal intention are somehow separated.

In the process of human communication, verbal interaction and verbal intention understanding represent the highest stage of communication. Vitality forms could be transmitted not only through observable gestures and actions, but also through verbal signs. According to the speaker’s attitude toward the listener, the listener can perceive the speaker mildly or rudely. Therefore, words that convey the form of vitality could allow the speaker to convey their inner mental state, and meanwhile, also allow the listener to understand the speaker’s affective feelings. Stern once asserted that infants could perceive the vitality forms of speech. For example, in mothers’ interactions with children, they often use a typical infant language for pronunciation. Specifically, the mothers can slow down the pronunciation of words to adapt their language to the children’s perceiving limits (Stern, 1998).

One article uses functional magnetic resonance imaging (fMRI) technology to study how people can recognize the inner state of others by listening to their speech (Di Cesare et al., 2016). Participants are asked to listen to action verbs in three different conditions: a human voice delivers the verb in a rude or gentle manner, a robot voice delivers the same verb but without vitality forms, and a scrambled version of the same verb is delivered by a human voice. Consistent with previous studies on encoding vitality forms, this study finds specific activation of the central part of the insula when listening to human voices which possibly convey specific vitality forms. Both posterior parts of the left inferior frontal gyrus and anterior parietal motion circuits are activated when hearing human and robotic voices, which are typically activated when observing and performing arm movements. In all those three cases, the superior temporal gyrus is activated on both sides. The conclusion of the study is that the central part of the insula is a key area for processing vitality forms, and it is capable of understanding vitality forms regardless of the way they are communicated or expressed. In a subsequent fMRI study, the same research team tries to determine that the DCI, which is involved in the verbal perception of vitality forms, also becomes active during the imaginary process of generating action verbs of different vitality forms (Di Cesare et al., 2017a). The experiment is based on the fMRI technique. Due to technical reasons, movement cannot be studied. So, researchers cannot directly study the form of vitality from the form of speech. The researchers use the motor imagination of the same motion verbs as a strategy to evaluate the possible activation of the insular cortex in the process of generating vitality forms. In the speech condition, the participants are asked to listen to or imagine themselves speaking softly or rudely. In the action condition, the subjects are asked to observe or imagine whether their actions were mild or rude. The results show that compared to the control condition, the DCI is activated in both the speech and action tasks. Indeed, it has also been shown that the circuits activated by motor imagination are the same as those activated during action execution, except for the primary motor cortex (Jeannerod, 1995).

### Vitality forms as grounding social cognition

Behavioral research has proved that even if there is no actual intention to act, the observation and indication of objects can even trigger an individual’s movement behavior. The possibilities of actions triggered by (actual or even virtual) object features are called “affordances” (Gibson, 1979; Bub and Masson, 2010). In other words, affordances are *possibilities for action*. In the category of emotion and affection, vitality forms reflect the changing mood or affective state of the agent in an ongoing dyadic interaction, so it might inevitably be affected by the socialized contextualization of social affordances. One usage of the concept of social affordances literally means possibilities for intersubjective interactions. Another usage refers to affordances that are shaped by sociocultural context, as in Gibson’s example of the postbox affordance of letter-mailing. It should be noted that one’s perception of the postbox as affording letter-mailing is also determined by this person’s previous sociocultural practice (imagine there is a country without a postal system) (cf. Gibson, 1979; de Carvalho, 2020; Borghi, 2021). In the first usage, social affordances can be seen as inherent properties of social surroundings that could constrain the possibilities of the actions executed by an agent. The second usage implies the malleable properties of social affordances.

For useful collections of existing resources, in the following passages of this subsection, actually we focus on the relevance of vitality forms and social affordances. In our view, both concepts in an essential way convey a sense of grounding relation between ground and its grounded things. The idea of social affordances implies a binary relation between affordances *per se* and their afforded (grounded) possible actions in a sociocultural context. In a similar way, vitality forms (as a ground) could also be regarded as *possibilities for action*, for the other two grounded higher dimensions of action, contents and intentions.^5^

Orban et al. (2021) advocate a promising neurophysiological framework of social cognition which can be firmly established on a rich source of social affordances of others.^6^ Roughly, the importance of vitality forms is for communicating with others through performing the style of action. In this view, vitality forms are always expressed toward the other agent within a dyad. Regarding the influence of others and the role of vitality forms in social interaction, several studies demonstrate that gentle or rude actions performed by the agent, however, affect the motor response of the receiver (cf. Di Cesare et al., 2017a, 2021b; Lombardi et al., 2021). In general, both vitality forms and social affordances are related to the modality of action, so to speak, yet separately focusing on different aspects; the former is on the kinematic signatures of action, while the latter is on the possibility of action. Vitality forms highlight the kinematic nature of the action, i.e., the how-dimension of action; social affordances stress on the objective properties of surroundings within which the agent has to be constrained.

The presence of a conspecific requesting gesture will change the way an individual interacts with an object. It has been shown that social affordances are activated in interactions between conspecific individuals premised on the social intention of feeding, which changes the sequence kinematics of reach-grasp tasks and placement (Ferri et al., 2010). This is related to the same sequence of pointing to inanimate targets. Subsequent research has shown that the social request (i.e., the gesture of requesting to open the mouth) made by the recipient is a prerequisite for activating social affordances. Specifically, even if the sequence that points to the same individual does not finalize eating, the social request to be fed will activate social affordances. Moving around the space of the same species without making any social requests has little effect on the sequence. A conspecific gaze is a necessary condition for social requests to effectively activate social affordances. In general, the control of motor sequences can be altered by the interaction between the actor and the receiver; it is the characteristic of interaction that the actor activates social affordances based on the social requirements generated by the receiver. The gaze of the recipient is a prerequisite for the validity of the social request (Ferri et al., 2011). One of the most important human abilities is to understand the behavior of other conspecifics.

Another series of studies explore the effect of sudden demands on the kinematics of pre-planned actions (Sartori et al., 2009). In experiment 1, in 80% of the trials, participants are asked to grab an object and put it in a container (no interference test). In the other 20% of the trials, the assistant sitting next to the participant accidentally extends their arm and opens their hand, as if asking for the object (interference trial). In the remaining 3 experiments, (a) the assistant is replaced by a machine, (b) the gestures made by the assistant do not imply social demands, and (c) the assistant’s gaze is unavailable. The results show that there is a kinematic change in the actions directed at the target only when the disturbance is a social request involving a human assistant. In contrast, there is no significant effect on kinematics when the interference is caused by a robot or by a human assistant performing a non-social gesture. These findings are discussed in the light of theories currently proposed to explain the influence of the social environment on action control. Another study aims to determine whether requested gestures and gaze direction are sufficient to infer communication intention in social contexts, by examining the influence of requested gestures and gaze direction on the kinematics of another individual’s arm movements (Innocenti et al., 2012). Research shows that social requests activate social affordances, which interfere with the control of sequences, and that gaze from potential recipients who hold the cup in their hands modulates the effectiveness of gestures. This paradigm, when applied to individuals with autism, could provide new insights into the nature of their impairments in social interaction and communication.

### Vitality forms as grounding social emotion

The category of emotion is different from that of cognition. In aiding the relevant discussions of vitality forms and their grounded social emotion, in this subsection, we turn to focus on a typical example of social emotion, that is, empathy. Empathy refers to the ability to put oneself in the situation of others in order to feel and understand both sides of feelings and cognitions. Vitality forms are not specific forms that could regulate the internal state of human movement behavior in a continuous manner. There might be a certain relationship between empathy and vitality forms. Twenty years ago, scholars propose the concept of the “mirror system,” which is a significant discovery (Rizzolatti et al., 1996; Gallese, 2001) in behavioral and social neuroscience. Its function is specifically manifested in that when the observer watches someone perform a goal-oriented action, the observer’s mirror neurons would fire in the same pattern, just as if the observer themself is performing the action. It can also imply that the observer shares a virtual experience of that actor’s experience. This has obvious meanings for understanding empathy and identification and response to artistic performance. How to capture the exact action characteristics of a specific individual in a certain way, and how to explain empathy and identification? Mirror neurons can suitably explain the “what” dimension of this behavior (goal-oriented). In addition, other mechanisms, such as intention detection centers, could explain the “why” dimension (intention and goal) (Ruby and Decety, 2001).

However, for recognition based on faithful imitation, people also need to understand the “how” dimension, the other person’s “dynamic movement characteristics,” i.e., their vitality forms (Hobson and Lee, 1999). Identification and internalization require greater complete immersion in the lively flowing experience of another person besides empathy. Without vitality forms, identification and internalization will act like rules of action rather than a state of perceived immersion. Heller and Haynal (1997) study therapeutic videotapes of high-risk patients who have repeated suicide attempts. The therapists could not predict who would attempt suicide again. Next, these two researchers and a panel of judges carefully examine the facial expressions of high-risk patients. Using Ekman and Friesen’s Facial Action Coding System, they are unable to forecast who would attempt suicide again. However, when they examine the facial expressions of the therapists, they could make important predictions about which patients would attempt suicide. This shows from another perspective that people are unaware of the role of vitality forms in recognition and empathy, a process that involves empathy and counter-empathy.

## Discussion

This article reviewed related currents on how to directly access other minds through vitality forms. Vitality forms are seen as a characterization of the action style based on the kinematics of actions, which might implicitly convey the internal state of the agent’s feelings and intentions. Vitality forms play a highly crucial role in basic interpersonal interactions (especially the parental–infant dyad). The effective perception of vitality forms allows people to directly know the attitudes of others simply through their actions, and even obtain relevant information about non-existent objects to which the pretended actions are directed. Similarly, the proper expression of the vitality forms enables others to understand people’s own internal state in the right way. The activation of the DCI when expressing and perceiving vitality forms strongly indicates that there is a neural mirroring mechanism of vitality forms (of actions) in the insula, which allows a person to express their own affective state and understand the affective state of others in the action.

However, current studies on vitality forms have focused on the identification of the characteristic dimension of mildness or rudeness of the action. It has shown that the idea of vitality forms involves the behavioral style of the action, so it does not only include mildness (or rudeness). In the single dimension of rudeness, actions can also be expressed as indecision or willingness, or cautiousness or recklessness. Future research perhaps could further expand and deepen this aspect. In addition, intention and behavioral style belong to two different dimensions of action. The brain mechanisms involved in perceiving action intention and vitality forms belong to two different areas. The former is the parietal frontal circuit, and the latter is the DCI. So, how can people perceive others’ intentions at the same time when they perceive the vitality forms characteristics of other people’s actions? What are the anatomical connections between the brain regions involved? Current studies are still forthcoming in this aspect. Finally, in the real-life social environment, actions are nested within the social context. In addition to arm movements, there are other important contextual cues, such as pre-existing information, the actor’s facial expressions, or even the observer’s interpersonal relationship with the actor. How do these contextual clues influence people to directly access other minds through the vitality forms of actions? Existing studies do not answer this question adequately, and this may be of great significance to the assessment of the perception of vitality forms of children with autism. After all, simple visual information may not be sufficient for children with autism to correctly encode vitality forms. Therefore, future studies might overcome this limitation by considering the use of different types of stimuli combinations.

**FIGURE 1:**
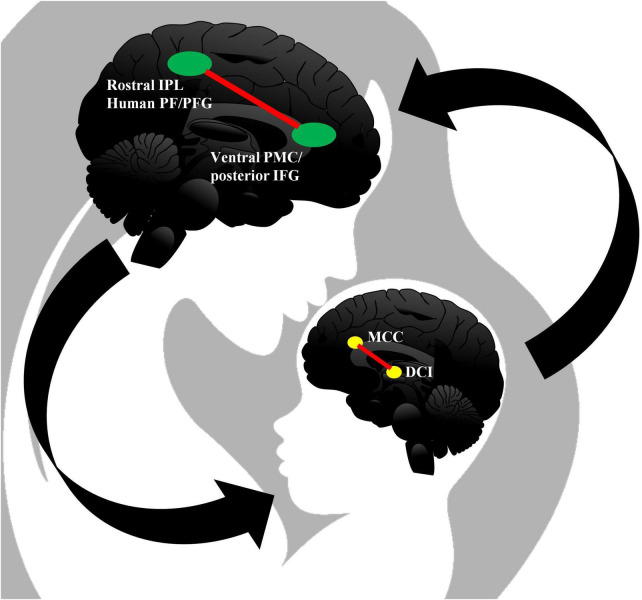
The neural bases of vitality forms (and human mirror neuron system, MNS) in a mother–infant communicative dyad. In the brain of the mother, the human MNS is composed of two parts: frontal MNS and parietal MNS. Generally, the anterior region with mirror neuron properties is located in the sub-frontal cortex, including the posterior frontal gyrus (IFG) and the adjacent ventral premotor cortex (PMC); the posterior region with mirror neuron properties is located in the rostral section of the inferior parietal lobe (IPL) and can be regarded as the human homolog of region PF/PFG in the macaque (cf. Pandya and Seltzer, 1982; Iacoboni and Mazziotta, 2007; Iacoboni, 2009; Bermúdez, 2020, p. 372). In the brain of the young baby, the picture presents a mirroring mechanism tuple (based on current trustworthy neuroimaging findings) of MCC and DCI, which can deal with observation and execution of vitality forms of action. Vitality forms occupy a new dimension apart from the what- and why-dimension of action, and ongoing actions *per se* convey enough in revealing the signatures of social perception. In a series of fMRI studies, researchers found selective activation of the DCI during both observation and execution of vitality forms (Di Cesare et al., 2014, 2017b). However, in one experiment, the MCC also showed activation (Di Cesare et al., 2020). In a subsequent fMRI study, the investigators employed the classical vitality forms paradigm, but eliminated the continuous style of movement by using jerky movements in a control condition to assess the role of the cingulate cortex in the processing of vitality forms. Participants performed two different tasks: observing a gentle or rude action and performing the same action. The results indicated that the MCC was strongly activated during action observation and execution, in addition to the insula (Di Cesare et al., 2021a). Besides, the neural bases of vitality forms should be incorporated into the larger MNS in the near future.

Traditional achievements of cognitive neuroscience hold that action comprehension is closely related to the mirror neuron system (MNS). However, recent studies have shown that a greater number of brain regions are involved in action comprehension. As shown above, previous studies imply that there is a certain link between the neural bases of the vitality forms and the mirroring mechanism of neurons. The right DCI, the main brain region activated in perceiving and expressing vitality forms, may be endowed with a mirror mechanism that translates sensory information about other people’s vitality forms into these forms of movement modules (Di Cesare et al., 2014, 2015). This may explain why children with ASD have difficulty in recognizing the vitality forms of other people’s actions; i.e., they have difficulty relying on their own misidentification of the corresponding vitality forms when observing others’ actions. It can also explain the difficulty of healthy children in recognizing the vitality forms of autistic children, that is, it is difficult for them to match the sensory modules of the motor kinematics observed in children with autism with their own brain processing and corresponding modules of vitality forms (see Figure 1). Di Cesare et al. (2021a) suggest that besides the DCI, the middle cingulate cortex (MCC) is also strongly activated during action observation and execution. This new finding is involved with a new how-dimension of action, by using jerky movements as a control condition.

So, up to the present, we get two trustworthy neural bases of vitality forms: DCI and MCC.^7^ In the end, in this article, for fulfilling the neural bases of vitality forms based on current neuroimaging studies by Rizzolatti, Di Cesare, and others, we highlight two areas, that is, DCI (actions executed in continuous condition) and MCC (actions executed in jerky condition), in Figure 1. Besides, the neural bases of vitality forms should be regarded as one part of the larger human MNS, since the former simply concerns the how-dimension of action rather than all dimensions involved in the social perception of dyadic interaction.

## Author contributions

DD and WC: conceptualization and supervision. QL and JZ: investigation. QL, DD, and JZ: writing of the original draft. QL and DD: reviewing and editing. All authors have read and agreed to the published version of the manuscript.

## Footnotes

1 We thank a referee for raising this issue.

2 We agree with Kemmerer that perceiving vitality forms is also modulated by “the situational context in which actions take place” (Kemmerer, 2021, p. 15).

3 For other usages of grounding relation in cognitive science, for example, Larsson (2018) distinguishes two interrelated senses of grounding: communicative grounding, primary pragmatic actions (like utterances) in dialogue ground all the other activities of verbal communications; symbol grounding, symbolized language (like words) is essentially grounded in the world.

4 We thank a referee for raising this issue.

5 We thank a referee for raising this issue.

6 For discussions of orthodox Gibsonian social affordances (see Rietveld et al., 2019; Baggs, 2021); for applications of social affordances in the context of Bayesian brain modeling (see Ramstead et al., 2016; Veissière et al., 2020).

7 Besides, another suggestion implies the potential role of the cerebellum (Ramsey et al., 2021). The role played by the cerebellum in social action comprehension is increasingly appreciated (Van Overwalle et al., 2019), and neurons with mirroring qualities are also present in the cerebellum (Molenberghs et al., 2012). Future research needs to elucidate the role of the cerebellum in social action comprehension. Individual action comprehension and learning may be closely related to mirror neurons, but the involvement of the cerebellum is essential when a series of actions are linked together in a temporal sequence. We imply that the relationship between the cerebellum and vitality forms is one of the future research directions.
